# Genomic Exploration of a Chitinolytic *Streptomyces albogriseolus* PMB5 Strain from European mantis (*Mantis religiosa*)

**DOI:** 10.3390/cimb46090554

**Published:** 2024-08-24

**Authors:** Vesselin Baev, Ivan Iliev, Elena Apostolova, Mariyana Gozmanova, Yana Hristova, Yanitsa Ilieva, Galina Yahubyan, Velizar Gochev

**Affiliations:** 1 Department of Molecular Biology, Faculty of Biology, University of Plovdiv, Tzar Assen 24, 4000 Plovdiv, Bulgaria; eapostolova@uni-plovdiv.bg (E.A.); mariank@uni-plovdiv.bg (M.G.); gyahubyan@uni-plovdiv.bg (G.Y.); 2 Department of Biochemistry and Microbiology, Faculty of Biology, University of Plovdiv, Tzar Assen 24, 4000 Plovdiv, Bulgaria; iziliev@uni-plovdiv.bg (I.I.); jhristova@uni-plovdiv.bg (Y.H.); vgochev@uni-plovdiv.bg (V.G.)

**Keywords:** *Streptomyces albogriseolus*, chitinases, genome sequencing, nanopore

## Abstract

The genus *Streptomyces* is renowned not only for its natural antibiotic production but also for its abundant chitinolytic enzymes, which break down stubborn chitin into chitooligosaccharides. Despite this, there have been limited studies utilizing whole-genome sequencing to explore the repertoire of chitin degradation and utilization genes in *Streptomyces*. A particularly compelling source of novel antimicrobials and enzymes lies in the microbiota of insects, where bacterial symbionts produce antimicrobials to protect against opportunistic pathogens and enzymes to adapt to the environment. In this study, we present the chitinolytic strain *Streptomyces albogriseolus* PMB5, isolated from the insectivorous *Mantis religiosa* (European mantis). Whole-genome sequencing revealed that PMB5 harbors a linear chromosome of 7,211,961 bp and a linear plasmid of 327,989 bp. The genome comprises 6683 genes, including 6592 protein-coding sequences and 91 RNA genes. Furthermore, genome analysis revealed 19 biosynthetic gene clusters covering polyketides, terpenes, and RiPPs, with 10 clusters showing significant gene similarity (>80%) to known clusters like antimycin, hopene, and geosmin. In the genome of *S. albogriseolus* PMB5, we were able to identify several antibiotic resistance genes; these included *cml* (resistance to phenicol), *gimA* (resistance to macrolides), *parY* (resistance to aminocoumarin), *oleC*/*oleD* (resistance to macrolides), *novA* (resistance to aminocoumarin) and *bla/blc* (resistance to beta-lactams). Additionally, three clusters displayed no similarity to known sequences, suggesting novel bioactive compound discovery potential. Remarkably, strain PMB5 is the first reported *S. albogriseolus* capable of thriving on a medium utilizing chitin as a carbon source, with over 50 chitin-utilizing genes identified, including five AA10 family LPMOs, five GH18 chitinases, and one GH19 chitinase. This study significantly enhances the genomic understanding of *S. albogriseolus*, a species previously underrepresented in research, paving the way to further exploration of the biotechnological potential of the species.

## 1. Introduction

Chitin, a highly prevalent renewable polysaccharide, is present in the exoskeletons and cell walls of numerous organisms. It comprises N-acetylglucosamine units linked by β-1,4 glycosidic bonds and typically forms crystal structures. Chitin, characterized by its insolubility in water, displays varying degrees of acetylation and molecular sizes up to several MDa. It has the propensity to interact with various organic and inorganic compounds. Primarily serving as a structural element, chitin is found in the cell walls of fungi and yeast, as well as in the exoskeletons of insects and crustaceans. While the intricate arrangement of chitin provides excellent resistance against both abiotic and biological degradation, it also poses challenges for advancing and exploiting biomass resources [1].

Chitinases are enzymes that degrade chitin by catalyzing the hydrolysis of β-1,4-glycosidic bonds, resulting in the production of chitooligosaccharides (COS), which are further broken down by chitobiases into N-acetylglucosamine (NAG). Chitinases can be classified into two main subgroups based on their mode of action: exochitinases and endochitinases [2]. They are categorized within the glycoside hydrolase families, GH18 and GH19. Chitinases belonging to the GH18 family are found in mammals, fungi, and bacteria, while those in the GH19 family are present in some bacteria and higher plants [3]. Furthermore, lytic polysaccharide monooxygenases (LPMOs) are metalloenzymes that cleave glycosidic bonds in crystalline chitin and facilitate the access of chitinases [4].

The production of chitinases from microorganisms is a versatile and valuable biotechnological process with a wide range of applications. One of the primary uses of chitinases is in the biological control of pests, particularly those with a chitinous exoskeleton. By breaking down the chitin in the exoskeleton, chitinases can effectively weaken and kill pests, providing an eco-friendly and sustainable solution to pest management. Beyond pest control, chitinases are also utilized in various biomedical applications. For instance, they have been shown to have antifungal properties, making them helpful in treating fungal infections [5]. Additionally, chitinases have been used to develop drugs for treating chitin-related diseases such as asthma and cystic fibrosis [6].

In the industrial sector, chitinases are used to degrade chitinous wastes, such as shrimp and crab shells, from the food industry. By breaking down these wastes, high-quality food and feed products can be produced, containing chitin COS, which have been shown to have numerous health benefits, including anti-inflammatory, antioxidant, and immune-enhancing properties [7]. However, research is needed to guarantee the bioactivity in processed foods and ensure their harmlessness, thus minimizing the risk of allergic reactions and allowing for the full utilization of edible insect species in the food industry [8]. Furthermore, chitinases are also used in the flavor enhancement industry to produce flavorful compounds to enhance the flavor of various food products. While the chitinases produced by microorganisms play a crucial role in converting shrimp and crab shell wastes, their molecular and ecological functions in industrial applications have not been thoroughly investigated [9].

*Streptomyces* species are extensively prevalent bacteria in soil and are vital as microbial agents in biomass breakdown within the carbon cycle. Actinomycetes, including *Streptomyces*, have developed intricate enzyme systems to extract soluble nutrients from chitin [10]. Examinations of genomes from different Streptomyces strains have unveiled numerous chitinase genes, which could potentially equip them to break down the various types of chitin they come across. Chitinases were identified to be produced by *Streptomyces venezuelae* P10 [11], *Streptomyces halstedii* [12], *Streptomyces viridificans* [13], *Streptomyces aureofaciens* [14], *Streptomyces* spp. ANU6277 [15], *Streptomyces* spp. M-20 [16], *Streptomyces* spp. DA11 [17], and *Streptomyces* spp. S-84 [18], among others. Recently, whole-genome sequencing technology has been extensively utilized to study the enzymatic repertoire of microorganisms that cause specific activities and effects. For example, an investigation into the genomic makeup of a *Streptomyces coelicolor* strain identified up to 13 potential chitinases [9]. Recent studies profiling the genomes of *Streptomyces californisus*, *Streptomyces parvulus*, and *Streptomyces diastaticus* strains also yielded information about various chitinase genes [19,20]. Hence, enhancing the reservoir of knowledge through whole-genome sequencing analyses of strains that produce chitinase will help in understanding the genetic foundation of chitin decomposition activity and the utilization of degraded chitin. Moreover, this will contribute to the discovery of novel sources of chitinases that can be harnessed in biotechnology and various industries.

Bacterial mutualists in the insect gut are indispensable to modulations of the host’s metabolism and to improving its digestion, thereby extracting maximum energy from the food consumed [21]. Therefore, the carnivorous insect hosts can be a valuable natural source of chitinolytic bacterial strains. In this study, we have screened and explored chitin-degrading streptomycetes within the gut microbiota of the carnivorous insect *Mantis religiosa* (European mantis). Such strains can potentially lead to the identification of new enzymes and antibiotics that could be used for biotechnological and medicinal purposes. The aim of the study is to explore the genomic context of the *Streptomyces albogriseolus* strain PMB5, which demonstrated significant chitinolytic activity, although the specific enzymes responsible for this activity were not identified. To determine the genes responsible for chitin degradation, our objective was to conduct whole-genome sequencing of the chitinase-producing *Streptomyces albogriseolus* strain PMB5 using nanopore sequencing technology. This approach facilitated the elucidation of the genomic architecture, enabling the identification of specific genes and their associated functions that are critical for chitin degradation.

## 2. Materials and Methods

### 2.1. Bacterial Strain Source and Sampling

The study was focused on insectivorous *Mantis religiosa*, which is primarily found in vegetation. Insect specimens were captured during two field expeditions in the city of Plovdiv and in the Strandzha Mountains in 2023. The Mantodea members’ behavioral analysis reports served as the basis for selecting the capture technique [22]. They are described as vicious predators who typically hunt by ambush. We used forceps to gather each specimen while ambushing prey and placed them in a sterile labeled container to prevent any potential surface contamination. Data recorded at each site included information, date, location, and host description. All specimens were kept alive at 4 °C until processed.

Specimens were surface pretreated by nebulization with 70% ethanol for 1 min to prevent external contamination. After drying, the specimens were treated for 30 s with a 1% bleach solution and a 0.1% Tween20 solution (Sigma, St. Louis, MO, USA). Finally, the specimens were rinsed 3 times using 1 mL PBS buffer for 10 s. We used a sterilized surgical scalpel to dissect each specimen during sample processing and then placed the entire digestive tract in 2 mL of sterile, phosphate-buffered saline (PBS) (Sigma, St. Louis, MO, USA). The resulting samples were homogenized in a vortex at 3000 rpm for 2 min [23].

### 2.2. Preparation of Colloidal Chitin

Colloidal chitin was prepared from shrimp chitin (Sigma, St. Louis, MO, USA) by gradually dissolving 10 g of chitin flakes with 150 milliliters of 10 N HCl and stirring overnight at 4 °C [24]. Chitin was formed into a colloidal suspension by gradually adding it to 2 L of water at a temperature range of 4–10 °C. The suspension was obtained through filtration using suction on a coarse filter paper. It was then washed by suspending it in approximately 5 L of distilled water. The washing process was performed three times until the suspension reached pH of 3.5. Following the aforementioned treatment, the unbound colloidal chitin was used as a substrate [25].

### 2.3. Screening of Chitinase-Producing Bacteria

Chitinase-producing bacteria were screened using the methodology outlined by Kuddus and Ahmad [26]. The Colloid Chitin Agar (CCA) medium used had the following concentrations (g L^−1^): K_2_HPO_4_, 0.7; KH_2_PO_4_, 0.3; NaCl, 5; MgSO_4_, 0.5; FeSO_4_, 0.001; ZnSO_4_, 0.001; MnSO_4_, 0.001; yeast extract, 0.5; agar, 15; and colloidal chitin at a concentration of 1% (*w*/*v*) (Sigma, St. Louis, MO, USA). After autoclaving at 121 °C for 15 min, the medium was cooled to 50 °C and amended with 100 mg L^−1^ nystatin and 50 mg L^−1^ nalidixic acid (Sigma, St. Louis, MO, USA).

All samples were serially diluted from 10^−1^ to 10^−5^ using a 1000 μL wide-bore tip and 100 μL of each dilution was inoculated using the spread plate method on plates with a CCA medium (Merck KGaA, Darmstadt, Germany). The colonies with a clean diameter against a cream-colored background were identified as presumptive chitinase-producing bacteria.

### 2.4. Secondary Screening for Chitin-Degrading Actinobacteria Strains

Actinomycete Isolation Agar (Merck KGaA, Darmstadt, Germany) was used to select actinobacteria. The screening was performed by spot-inoculating all the chitin-degrading bacterial isolates using toothpick heads and incubating them aerobically at 28 °C. The clearance zone due to chitin hydrolysis was recorded at 7 and 14 days. A strain identification number was given to the bacterial isolates exhibiting distinct actinomycete morphology and generating clear zones exceeding 0.5 cm on their own.

The presumptive assignment of the bacterial isolates was carried out by the methods suggested in Bergey’s Manual of Systematic Bacteriology [27]. Isolates were identified based on a polyphasic method, including morphological and cultural characteristics. Morphology of the areal mass and substrate mycelium were observed on AIA and were used for classification and differentiation, as described by Antido et al. [28]. The morphological characteristics consist of the shape, profile, surface, edge, color of aerial mycelia, color of the substrate mycelia, and the Gram stain appearance and spore chain morphology under 1000× magnification.

### 2.5. DNA Extraction, Sequencing Library Generation, Sequencing, and Assembly

DNA from the bacterial isolate was extracted utilizing the QIAamp DNA Microbiome Kit (QIAGEN, Hilden, Germany). DNA quantity and quality were assessed using Qubit 4 fluorometer (Thermo Fisher Scientific, Waltham, MA, USA) and agarose gel electrophoresis, respectively.

To prepare long-read Oxford Nanopore Technology (ONT) libraries, the Ligation Sequencing Kit SQK-RBK114 (Oxford Nanopore Technologies, Oxford, UK) was utilized with 200 ng of total DNA following the manufacturer’s protocol. Subsequently, the prepared library underwent sequencing on a MinION device using an R10 flow cell (Oxford Nanopore Technologies, Oxford, UK). The updated default manufacturer protocol was used for the kit SQK-RBK114 (https://community.nanoporetech.com/docs/prepare/library_prep_protocols/rapid-sequencing-gdna-barcoding-sqk-rbk114/v/rbk_9176_v114_revm_27nov2022, accessed on 23 July 2024). Base calling and quality control were conducted offline using Guppy v6.5.7 (Oxford Nanopore Technologies, Oxford, UK) (https://community.nanoporetech.com/docs/prepare/library_prep_protocols/Guppy-protocol/v/gpb_2003_v1_revax_14dec2018/guppy-software-overview, accessed on 23 July 2024), and adapter trimming was performed using Porechop v.0.2.4 with default parameters (https://github.com/rrwick/Porechop, accessed on 23 July 2024).

De novo assembly of the sequenced reads was carried out using Flye v2.9.2 with default parameters, excluding reads shorter than 1000 base pairs. Assembly polishing was accomplished using the Racon v1.4.21 (https://github.com/isovic/racon, accessed on 23 July 2024) and Medaka v1.8.1 (https://github.com/nanoporetech/medaka, accessed on 23 July 2024) tools. The quality of the assembled genome sequence was assessed using the CheckM v1.1.6 tool [29]. Finally, a circular genome map was generated from the single chromosome contig using the Circus v.0.69 and Proksee tool v.1.0 (https://proksee.ca/, accessed on 23 July 2024) [30,31].

### 2.6. Genome-Based Strain Identification

The genome sequence was subjected to MultiLocus Sequence Typing (MLST) analysis at PubMLST, https://pubmlst.org, accessed on 23 July 2024) [32]. To identify the bacterial species, the ANI (Average Nucleotide Identity) of the isolate was calculated using FastANI [33]. Currently, six submitted genomes of *S. albogriseolus* (also known as *S. viridodiastaticus*) are publicly accessible and used in ANI analysis. Subsequently, a phylogenetic tree based on the whole-genome sequence was constructed using the Type (Strain) Genome Server (TYGS) available at https://tygs.dsmz.de/ (accessed on 23 July 2024) [34].

### 2.7. Genome Annotation

The genome sequence of PMB5 was submitted to NCBI Genomes, and accession numbers were allocated. Following this, the resulting GenBank file underwent annotation via the Rapid Annotations using the Subsystems Technology (RAST) webserver [35]. Furthermore, functional annotations were conducted utilizing the KEGG database and BlastKOALA toolv3.0 [36]. The outcome of the tools was merged to produce more enhanced annotations.

To assess antimicrobial resistance (AMR) genes, the PMB5 genome was scanned using the Abricate tool with default parameters against the Comprehensive Antibiotic Resistance Database (CARD) [37], MEGARes DB [38,39]. Additionally, AMR annotation was enriched with entries from the BlastKOALA tool. Carbohydrate-Active Enzyme (CAZyme) gene analysis was performed using the dbCAN3 server and a Hidden Markov Model (HMM) profile retrieved from the dbCAN3 HMMdb database (version 7.0) [40,41]. The genome-wide identification, annotation, and analysis of secondary metabolite biosynthesis gene clusters were done using AntisMASH 7.0 [42].

### 2.8. Data Availability

The genome sequence of the strain PMB5 was submitted to GenBank under the accession numbers CP151652 and CP151653 (plasmid) and BioProject PRJNA1099411.

## 3. Results and Discussion

### 3.1. Presumptive Identification of the Chitinase-Producing Bacteria

**Figure 1:**
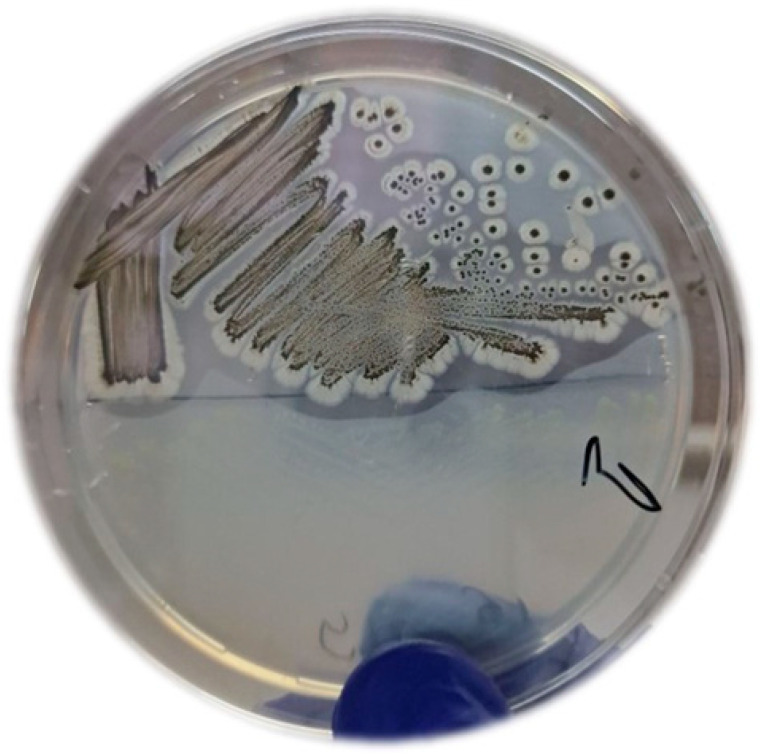
Morphology of isolate PMB5 on 1% colloid chitin agar (CCA) medium and clearance zone produced due to chitin hydrolysis.

The digestive tracts of 10 specimens of *Mantis religiosa*, all collected within the limits of the city of Plovdiv, Bulgaria, yielded a total of 18 different chitinolytic bacterial strains. Among the collected strains, only PMB5 produced a significant zone of clearance (>0.5 cm) and exhibited a typical-for-the-genus *Streptomyces* morphology (Figure 1).

Visual observation of the morphological and microscopic characteristics of the PMB5 strain was identified as the genus *Streptomyces*. The colonies were slow-growing, aerobic, chalky, and heaped. The aerial mass was classified according to Bergey’s manual of systematic bacteriology as gray (Gy), with ivory (Iv)-colored substrate mycelium. In addition, all colonies possessed a typical earthy odor. The strain PMB5 was acid-fast negative and Gram-stain positive, with a catalase-positive and oxidase-negative reaction. It did not produce water-soluble pigments. After the preliminary identification, the strain PMB5 was subjected to genome-based sequencing to reveal its full genomic landscape and suggest possible biotechnological applications.

### 3.2. In Silico Genomic Landscape

#### 3.2.1. Genome Overview and Species Identification

We utilized nanopore ONT sequencing to explore the genomic context of the bacterial strain. Nanopore sequencing offers distinct advantages over traditional next-generation sequencing (NGS) methods, notably in generating longer reads, which enhance the resolution of complex genomic regions and repetitive sequences. Particularly, ONT facilitates the simpler assembly of bacterial genomes. Recent advancements, especially with R10 flow cells, have improved read lengths, reduced error rates, and enhanced sequencing accuracy. Software-based calling algorithms have further refined ONT sequencing data. These advancements are progressively enabling the standalone use of ONT, reducing dependence on additional Illumina data.

**Figure 2:**
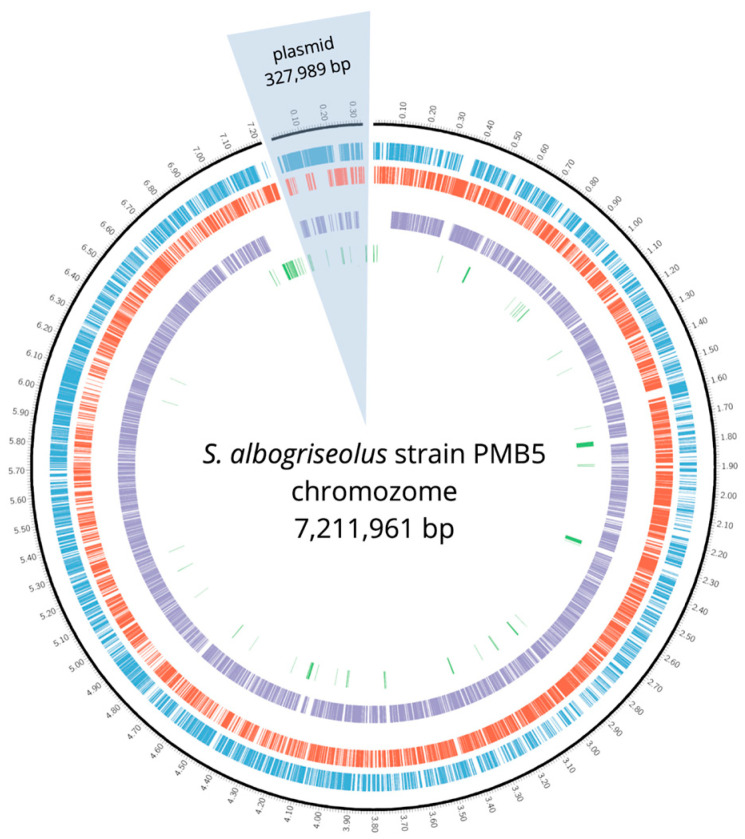
Map of the *S. albogriseolus* strain PMB5 genome visualized using the Proksee tool—bacterial chromosome (linear) and plasmid (linear). Forward genes are depicted in blue, reverse genes in red, core-genes in purple and strain-specific genes in green.

The assembled genome of PMB5 has a linear chromosome measuring 7,211,961 bp in length, boasting a GC content of 72.55% (BioProject accession: PRJNA1099411) (depicted in Figure 2). Additionally, it contains a linear plasmid spanning 327,989 bp with a GC content of 73.95%.

**Figure 3:**
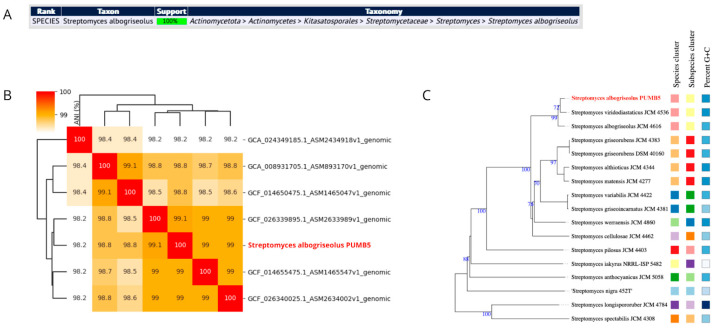
Specific Confirmation of the PMB5 genome: (**A**) PubMLST results; (**B**) whole-genome phylogenetic tree generated by TYGS database; and (**C**) ANI heatmap calculated between the PMB5 genome and publicly available genomes at NCBI.

MLST is a widely embraced technique for identifying and typing microbial organisms [43]. It entails analyzing the sequences of multiple housekeeping genes, culminating in a distinctive allelic profile for the microorganisms. MLST is regarded as highly accurate, facilitating easy comparison and exchange of results across various studies [44]. Notably, PubMLST analysis of the PMB5 genome displayed full support for the *S. albogriseolus* species (Figure 3A). The assembled genome similarity was assessed via ANI, comparing the PMB5 strain genome with the six complete *S. albogriseolus* genomes accessible on NCBI (as illustrated in Figure 3B). The ANI values for the PMB5 strain ranged from 98.2% to 99.1%, which falls within the species threshold of >95–96% ANI, recently adopted criterion for improving the taxonomic assignment in prokaryotic genomes [33]. Additionally, phylogenomic analysis through genome–genome comparisons in TYGS revealed that the PMB5 strain clusters with other representative strains in the database (as depicted in Figure 3C). MOB-Typer analysis revealed that PMB5 harbors one non-mobilizable linear plasmid. The genome statistics closely resemble those from a recent comprehensive study of *S. albogriseolus* strain LBX-2, the sole genome in NCBI classified as complete [45].

#### 3.2.2. Genome Annotation

**Figure 4:**
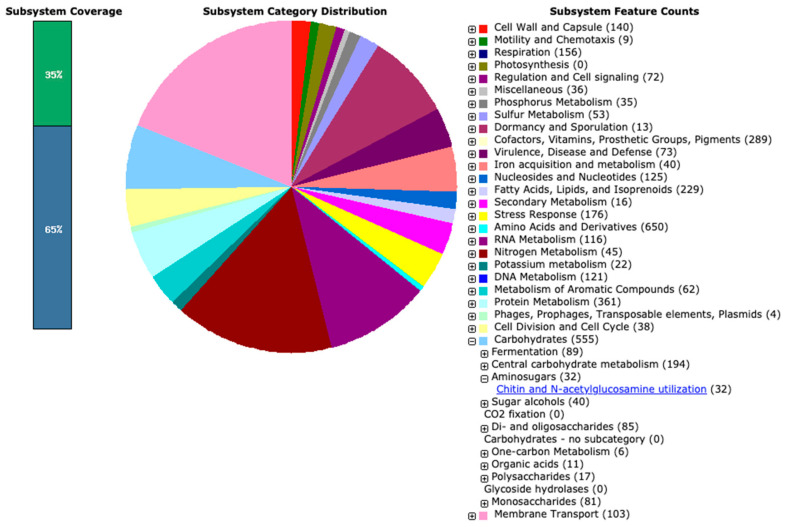
Distribution of *S. albogriseolus* PMB5 subsystem gene functions. The pie chart shows the count of each subsystem feature and subsystem coverage.

To explore the genomic landscape of the newly assembled genome of the PMB5 strain, we employed the RAST server and complemented the annotation with BlastKOALA results. A total of 6683 genes were identified, comprising 6592 protein-coding sequences (CDS) and 91 RNA genes (70 tRNAs, 18 rRNAs, and 3 ncRNA). Among the predicted CDS, 4793 genes (72.71%) were functionally annotated, while 1799 genes (27.29%) remained hypothetical/unknown. RAST analysis revealed the involvement of proteins in 439 subsystems (Figure 4). Supplementary Data Table S1 contains a detailed breakdown of the RAST and BlastKOALA annotations. Notably, these genes were predominantly associated with various protein families, including amino acids and derivatives (650), carbohydrates (555), protein metabolism (361), cofactors, vitamins, prosthetic groups, pigments (289), fatty acids, lipids, and Isoprenoids (229), among others. The genus *Streptomyces* is known for its ability to produce a wide variety of secondary metabolites, including antibiotics, which enables them to adapt to diverse environmental conditions [46]. In this context, numerous genes related to stress response (176), the metabolism of aromatic compounds (62), sulfur metabolism (53), nitrogen metabolism (45), phosphorus metabolism (35), potassium metabolism (22), secondary metabolism (16), resistance to antibiotics and toxic compounds (51), and more, were identified. Regarding chitin and N-acetylglucosamine utilization, the RAST tool identified 32 genes.

In the genome of *S. albogriseolus* PMB5, we were able to identify several antibiotic resistance genes (ARG) harbored exclusively in the chromosome; these included *cml* (resistance to phenicol), *gimA* (resistance to macrolides), *parY* (resistance to aminocoumarin), *oleC*/*oleD* (resistance to macrolides), *novA* (resistance to aminocoumarin) and *bla*/*blc* (resistance to beta-lactams). The ARG in *Streptomyces* species are a significant concern due to their potential to contribute to the development of antibiotic resistance in other organisms, including pathogenic bacteria, through the process of horizontal gene transfer [47]. However, according to McDonald and Currie [48] such transfers are surprisingly rare and are part of the evolution of the bacterial lineages. On the other hand, the genes established in the present study are frequently identified in *Streptomyces* as part of its natural self-resistance mechanisms. These resistance genes are widely detected in soil [49] and clinical specimens [50], suggesting a high prevalence of these ARG in those environments. Furthermore, current research on *Streptomyces* ARG indicates that the phenotypes can be sensitive to environmental filters and locally adapted, implying that the resistance of each strain is the result of local selection mediated by species interaction [51].

*Streptomyces* species are known for their ability to produce a wide range of secondary metabolites, including antibiotics and other bioactive compounds. These compounds are encoded by biosynthetic gene clusters (BGCs) often located on linear plasmids [52]. Some examples of *Streptomyces* species with many biosynthetic clusters include *S. rimosus*, which has a giant linear plasmid pSCL4 carrying the biosynthetic gene cluster for neocarzinostatin. Other *Streptomyces* species, such as *S. carzinostaticus*, *S. globispora*, and *S. atroolivaceus*, also have their antibiotic clusters located on giant linear plasmids [53]. These plasmids play a significant role in antibiotic production evolution and transfer among *Streptomyces* species through horizontal gene transfer. In addition to linear plasmids, *Streptomyces* species also have a large number of secondary metabolite biosynthetic gene clusters (SM-BGCs) that may encode natural products (NPs). However, most of these BGCs are silent under standard laboratory conditions, and activation of these silent BGCs is essential for current natural product discovery research [54].

Genome mining has been used to identify and activate novel biosynthetic gene clusters in *Streptomyces* species. For example, genome exploration of *Streptomyces collinus* Tü 365 revealed 32 gene clusters encoding the biosynthesis of diverse secondary metabolites, indicating the enormous biosynthetic potential of this strain [55]. Identifying and activating these gene clusters can lead to the discovery of new bioactive compounds with potential applications in various fields such as medicine, agriculture, and biotechnology.

Based on our investigation into the genome, we identified a total of 19 potential biosynthetic gene clusters (Table 1). These clusters encompass a variety of compound classes, including four polyketides, four terpenes, four RiPPs, etc. Notably, 10 of these clusters exhibited a significant resemblance, with over 80% gene similarity, to antymicyn, hopene, citrylassin D, geosmin, streptamidin, albaflavenone, aborycin, desferrioxamin B/E, ectone, and alkylresorcinol gene clusters. The PMB5 strain under examination showcased a diverse array of gene clusters linked to the biosynthesis of various antimicrobial and antifungal compounds, such as antimycin, informatipeptin, paulomycin, citrulassin D, streptamidin, aborycin, naphthomycin A, and albaflavenone, collectively bolstering its defense mechanisms against microbial threats [56,57,58]. Additionally, gene clusters encoding desferrioxamins B and E indicate an additional strategy for combating microbial invaders through iron regulation and have been used in the pharmaceutical industry. The strain’s genetic repertoire extends to compounds like antimycin, albaflavenone, and aborycin, which exhibit potential antifungal properties, further enhancing its ability to counter fungal pathogens [59]. Antimycin also demonstrates an anticancer activity, making it a promising candidate for developing anticancer agents. Its biological effects are easily identifiable through screening processes. However, despite its potential, only six strains known to produce antimycin have been experimentally confirmed [60]. This diverse range of antimicrobial and antifungal compounds synthesized by the PMB5 strain highlights its promising potential as a valuable source of bioactive molecules with significant pharmaceutical applications. Two gene clusters showed low similarity (<30%) to paulomycin, naphthomycin A gene clusters.

Interestingly, three potential gene clusters did not demonstrate conservation compared to any known cluster. Notably, two of these clusters contain lanthionine biosynthesis cyclase *LanC* and lanthionine biosynthesis protein *LanB* genes for putative lanthipeptide class I production. These clusters are located at the end of the linear chromosome, where the genome shows high concentration of species- and/or strain-specific genes. This supports the idea that the terminal regions are usually hotspots for genetic diversity, enabling different strains to adapt and evolve unique traits [61,62]. These concealed secondary metabolite biosynthetic gene clusters (with low or no similarity) suggest the possibility of discovering new antibiotics or other bioactive natural compounds from the strain PMB5.

Linear plasmids of considerable size are prevalent among actinobacteria and play a significant role in secondary metabolite production, including antibiotics. BLAST analysis also showed only around 40% coverage similarity of the plasmid to other *Streptomyces* plasmids sequences. In the strain PMB5, the plasmid carries biosynthetic clusters resembling kosinostatin (62%) and lagmysin (80%). Significantly, the kosinostatin-like cluster spans the entire plasmid, comprising 176 genes. Moreover, the similarity percentage suggests that further in-depth exploration is warranted, as this plasmid cluster may harbor unexploited potential for discovering novel natural products. Kosinostatin is a secondary metabolite produced by certain strains of *Streptomyces* that have been shown to have antimicrobial and antiproliferative activities [63]. These compounds have potential as lead molecules for developing new antibiotics and anticancer agents, and further research is needed to understand their mechanisms of action and potential therapeutic applications fully.

**Table 1:** Predicted secondary metabolite biosynthesis gene clusters and their location in the genome of *S. albogriseolus* PMB5.

Location	Type	From	To	Most Similar Known Cluster	Class	Similarity
chromosome	lassopeptide, T1PKS, NRPS	468	70,358	antimycin [60]	NRP + Polyketide	100%
	NRPS, RiPP-like	323,156	401,724	informatipeptin [64]	RiPP:Lanthipeptide	57%
	terpene	759,678	786,375	hopene [65]	Terpene	92%
	lassopeptide	1,136,682	1,159,270	citrulassin D	RiPP	100%
	NI-siderophore	1,206,990	1,238,161	paulomycin [57,66]	Other	13%
	terpene	1,367,859	1,390,015	geosmin [67]	Terpene	100%
	RiPP-like	1,413,136	1,424,449			
	LAP	1,637,023	1,659,540	streptamidine [68]	RiPP:Other	100%
	terpene	2,409,081	2,430,166	albaflavenone [69]	Terpene	100%
	RRE-containing	2,554,459	2,575,655	naphthomycin A [58]	Polyketide	9%
	lassopeptide	3,284,036	3,306,529	aborycin [70]	RiPP	100%
	NI-siderophore	4,613,401	4,643,173	desferrioxamin B/desferrioxamine E [71]	Other	100%
	ectoine	5,636,782	5,647,180	ectoine	Other	100%
	T2PKS	6,450,985	6,523,494	spore pigment [72]	Polyketide	83%
	terpene	6,710,643	6,734,757	carotenoid [73]	Terpene	54%
	T3PKS	6,948,679	6,989,752	alkylresorcinol [74]	Polyketide	100%
	lanthipeptide-class-i	7,026,827	7,051,355			
	lanthipeptide-class-i	7,129,548	7,153,689			
plasmid	transAT-PKS, NRPS, PKS-like, ectoine, T2PKS, hglE-KS, lanthipeptide-class-i, lanthipeptide-class-ii	26,123	301,355	kosinostatin [63]	NRP + Polyketide	62%
	lassopeptide	304,861	327,519	lagmysin	RiPP	80%

#### 3.2.3. The Repertoire of CAZymes and Genes for Chitin Utilization in the PMB5 Genome

CAZymes are important for the ecological and biotechnological relevance of *Streptomyces* bacteria. *Streptomyces* genomes demonstrate high levels of variability not only in their secondary metabolite biosynthetic gene clusters (smBGCs), but also with their CAZyme repertoire [75]. This emphasizes the need for strain-specific genomic mining to understand the potential of *Streptomyces* species fully.

The analysis of the inherent modules within CAZymes primarily from gene sequences serves to evaluate and determine an organism’s capability to produce enzymes that degrade complex carbohydrates. Combining the outputs of the three provided methods within the dbCAN3 tool, the automated CAZyme annotation aims to achieve the most accurate results possible. Only those CAZymes detected by at least two methods are included and presented in Table 2. We detected 14 enzymes with auxiliary activity (AA), 41 enzymes with carbohydrate-binding module (CMB), 26 carbohydrate esterases, 122 glycoside hydrolases, and 55 glycosyl transferases.

#### 3.2.4. Genetic Repertoire of Chitin Breakdown and Utilization Genes in PMB5

To our knowledge, there have been no reports on the ability of *Streptomyces* species *S. albogriseolus* to thrive on a medium utilizing chitin as a carbon source. *S. albogriseolus* strain PMB5 has emerged as a promising candidate due to its demonstrated chitinolytic activity. Understanding the enzymatic profile involved in chitin utilization and metabolism is crucial for biotechnological applications and ecological and environmental reasons. Thus, delving deeper into the enzymatic capabilities of the strain PMB5 and similar organisms holds significant promise for scientific advancement and practical applications in various fields. The integration of wet-lab chitin assay screening with dry-lab complete genome annotation of PMB5 significantly enhances our comprehension of chitin breakdown and metabolism within this strain and within this *Streptomyces* species, which has so far received limited attention in research. As we noted, a characteristic of the PMB5 strain is its proficient ability to break down chitin and utilize it as a growth source. Within the PMB5 genome, multiple genes are coding for specific enzymes engaged in chitin breakdown and utilization (Table 3).

The direct breakdown of chitin might occur through a coordinated effort involving LPMOs, chitinases, and N-acetylhexosaminidase, resulting in the production of chitooligosaccharides. Chitooligosaccharides are transported into the cell in the *Streptomyces* system through the following three transporter systems: the ABC transporters NgcEFG and DasABC [76,77], as well as the phosphotransferase system (PTS) [78]. We have identified genes from all three transporter systems.

**Figure 5:**
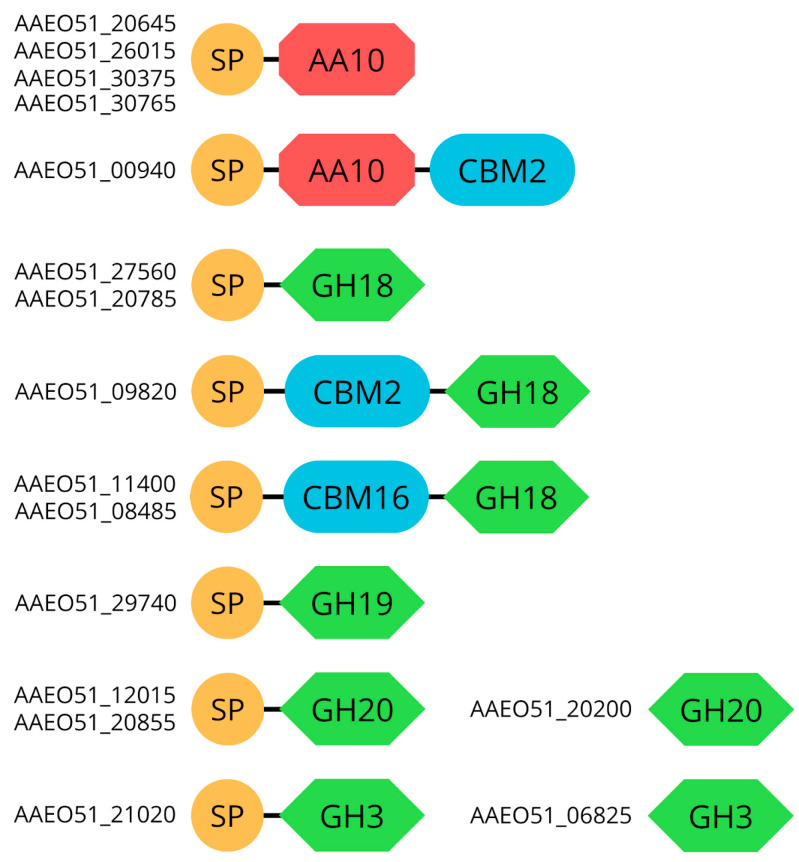
Structural domain organization of predicted CAZymes involved in the direct hydrolysis of chitin. SP: signal peptide; GH: glycoside hydrolase; CBM: carbohydrate-binding module; AA: auxiliary activities.

The PMB5 genome contains five LPMOs (Table 3), which are all part of the AA10 family. Furthermore, the genome harbored five GH18 chitinases and one GH19. Chitinases belonging to family 18 are widespread among various organisms, but those of family 19 are found almost only in higher plants. For Actinobacteria, the distribution of family 19 chitinase was systematically studied, and it was suggested that family 19 chitinases of *Streptomyces* species were acquired from plants by horizontal gene transfer. Four of the LPMOs are single domains and one contains two domains, AA10 + CBM2 (AAEO51_00940) (Figure 5). The presence of CBM2 within AA10 members underscores its dual role in facilitating enzyme-targeting to substrates and extending the duration of LPMO activity [79]. Regarding chitinases GH19 and two GH18 are single domains, whereas three GH18 have two domains—CBM16 + GH18 (AAEO51_08485), CBM2 + GH18 (AAEO51_09820), CBM16 + GH18 (AAEO51_11400) (Figure 5). Like LPMOs, chitinase genes that consist of either CBM2 or CBM16 domains may enhance the binding affinity to chitin substrates. All chitinases and LPMO enzymes possess signal peptides in their sequences. This feature is highlighted in the genomic analysis of chitin-degrading genes, where the presence of these domains in chitinases is associated with an increased ability to bind to chitin substrates, potentially enhancing the efficiency of chitin degradation processes [20].

**Figure 6:**
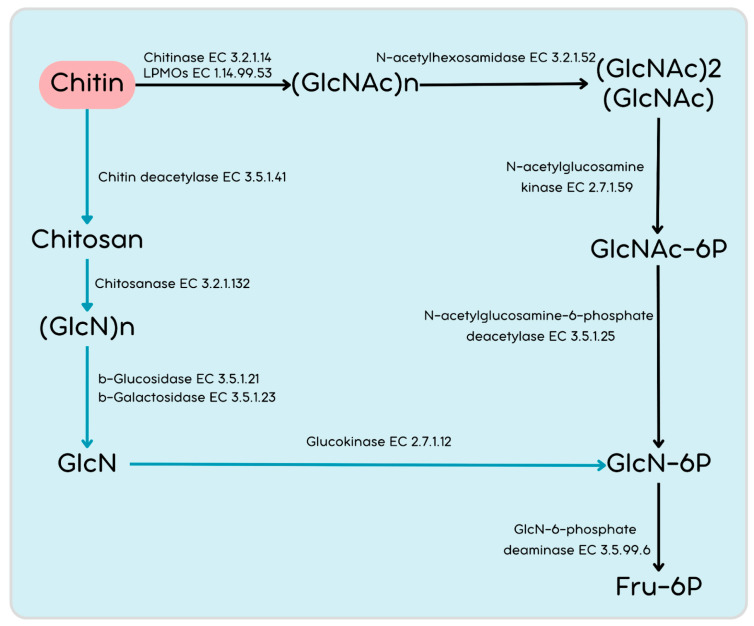
Proposed pathways of chitin degradation and utilization in *S. albogriseolus* PMB5.

The PMB5 genome encodes N-acetylhexosaminidases classified into the GH20 (AAEO51_12015, AAEO51_20200, AAEO51_20855) and GH3 (AAEO51_06825, AAEO51_21020) families, which play important role hydrolyzing chitobiose to GlcNAc (Figure 6). Furthermore, GlcNAc may undergo phosphorylation to GlcNAc-6P by N-acetylglucosamine kinase (the PMB5 genome encodes five genes). GlcNAc-6P can be deacetylated to GlcN-6P by the N-acetylglucosamine-6-phosphate deacetylase on the downstream pathway. Moreover, GlcN-6P can be deaminated to fructose-6P by GlcN-6-phosphate deaminase. An alternative putative pathway for chitin utilization involves chitosan, employing enzymes, such as polysaccharide deacetylasesas, from the CE4 family (putative chitin deacetylases) and chitosanases (GH46 or GH75), which are also present in the PMB5 genome.

## 4. Conclusions

This study provides valuable insights into the genomic landscape of *Streptomyces albogriseolus* strain PMB5, isolated from the digestive tract of *Mantis religiosa*. Whole-genome sequencing and analysis revealed a linear chromosome of 7.2 Mb and a linear plasmid of 328 kb. The genome harbors 19 biosynthetic gene clusters, including several with the potential for novel antimicrobial compound discovery). These clusters encompass a variety of compound classes, including four polyketides, four terpenes, and four RiPPs, etc. Interestingly, three potential gene clusters did not demonstrate conservation compared to any known cluster. Notably, two of these clusters contain lanthionine biosynthesis cyclase *LanC* and lanthionine biosynthesis protein *LanB* genes for putative lanthipeptide class I production. Such concealed secondary metabolite biosynthetic gene clusters (with low or no simi-larity) suggest the possibility of discovering new antibiotics or other bioactive natural compounds from the strain PMB5. In the genome of *S. albogriseolus* PMB5, we were able to identify several antibiotic resistance genes; these included cml (resistance to phenicol), *gimA* (resistance to macrolides), *parY* (resistance to aminocoumarin), *oleC*/*oleD* (resistance to macrolides), *novA* (resistance to aminocoumarin), and *bla*/*blc* (resistance to beta-lactams). Notably, PMB5 possesses an extensive repertoire of CAZymes and genes involved in chitin degradation and utilization, including chitinases, LPMOs, and related enzymes. The PMB5 genome contains five LPMOs, all part of the AA10 family. Furthermore, the genome harbored five GH18 chitinases and one GH19. This genomic exploration enhances our understanding of *S. albogriseolus* and its chitinolytic capabilities, opening avenues for future research in biotechnology, environmental science, and industrial applications.

## Figures and Tables

**Table 2:** Detected CAZymes and their count in the PMB5 genome.

CAZy Function Class	CAZy Family (No.)
Auxiliary activity	AA0 (1), AA10 (5), AA3 (2), AA4 (1), AA5 (1), AA7 (4)
Carbohydrate-binding module	CBM13 (10), CBM16 (2), CBM2 (10), CBM25 (2), CBM3 (1), CBM32 (3), CBM35 (2), CBM4 (1), CBM48 (4), CBM6 (2), CBM61 (1), CBM67 (2), CBM92 (1)
Carbohydrate esterases	CE1 (2), CE12 (2), CE14 (3), CE19 (1), CE3 (3), CE4 (11), CE7 (2), CE8 (1), CE9 (1)
Glycoside hydrolases	GH0 (2), GH1 (6), GH10 (4), GH11 (3), GH12 (2), GH13 (20), GH146 (1), GH15 (4), GH16 (2), GH17 (3), GH18 (5), GH19 (1), GH2 (5), GH20 (3), GH23 (7), GH25 (3), GH27 (1), GH3 (5), GH30 (1), GH31 (2), GH35 (1), GH36 (1), GH38 (1), GH39 (1), GH4 (2), GH42 (3), GH43 (3), GH46 (3), GH5 (4),GH51 (1),GH52 (1), GH6 (4), GH64 (2), GH65 (3),GH67 (1), GH74 (1), GH75 (1), GH77 (1), GH81 (2), GH84 (1), GH87 (2), GH92 (2), GH95 (1)
Glycosyl transferases	GT0 (1), GT1 (3), GT2 (20), GT20 (1), GT28 (2), GT35 (1), GT39 (1), GT4 (15), GT51 (4), GT76 (2), GT81 (1), GT83 (1), GT83 (1), GT84 (1), GT87 (1), GT9 (2)

**Table 3:** Genes in PMB5 genome involved in chitin utilization.

Gene(s) ID in PMB5 Genome	EC	Activity
AAEO51_08485 AAEO51_09820 AAEO51_11400 AAEO51_27560 AAEO51_20785	EC: 3.2.1.14	Chitinase (GH18)
AAEO51_29740	EC: 3.2.1.14	Chitinase (GH19)
AAEO51_02230 AAEO51_02920 AAEO51_05575 AAEO51_06195 AAEO51_20110 AAEO51_27435 AAEO51_12580 AAEO51_12585 AAEO51_21230	EC: 3.5.1.41/EC: 3.1.1.72	Chitin deacetylase/Acetyl xylan esterase (CE4, polysaccharide deacetylases)
AAEO51_01075 AAEO51_02335 AAEO51_02750 AAEO51_04200 AAEO51_04895 AAEO51_08435 AAEO51_22480 AAEO51_29405 AAEO51_31010 AAEO51_31015 AAEO51_31560	EC: 3.2.1.23	β-Galactosidase
AAEO51_00800 AAEO51_02890 AAEO51_20790 AAEO51_23445 AAEO51_31395	EC: 3.2.1.21	β-Glucosidase
AAEO51_24520 AAEO51_29530	EC: 3.2.1.132	Chitosanase (GH46/GH75)
AAEO51_26300	EC: 2.7.1.12	Glucokinase
AAEO51_12700 AAEO51_20840	EC: 2.6.1.16	GlcN-fructose-6-phosphate aminotransferase (isomerization)
AAEO51_12720	EC: 5.4.2.10	Phosphoglucosamine mutase
AAEO51_00475 AAEO51_10410	EC: 3.5.99.6	GlcN-6-phosphate deaminase
AAEO51_19315 AAEO51_19735	EC: 2.3.1.157/EC: 2.7.7.23	GlcN-1-phosphate N-acetyltransferase
AAEO51_17955	EC 3.5.1.25	N-acetylglucosamine-6-phosphate deacetylase
AAEO51_21430 AAEO51_28730 AAEO51_01145 AAEO51_17960	EC 2.7.1.59	N-acetylglucosamine kinase
AAEO51_21020 AAEO51_06825 AAEO51_12015 AAEO51_20200 AAEO51_20855	EC 3.2.1.52	N-acetylhexosaminidase
AAEO51_00940 AAEO51_20645 AAEO51_26015 AAEO51_30375 AAEO51_30765	EC: 1.14.99.53/1.14.99.54	Lytic polysaccharide monooxygenases (LPMOs)

## Data Availability

Data is contained within the article.

## Supplementary Materials

The following supporting information can be downloaded at: https://www.mdpi.com/article/10.3390/cimb46090554/s1, Table S1: Genome annotation of PMB5.
